# Burden and Risk Factors for Cold-Related Illness and Death in New York City

**DOI:** 10.3390/ijerph15040632

**Published:** 2018-03-30

**Authors:** Kathryn Lane, Kazuhiko Ito, Sarah Johnson, Elizabeth A. Gibson, Andrew Tang, Thomas Matte

**Affiliations:** 1 Bureau of Environmental Surveillance and Policy, New York City Department of Health and Mental Hygiene, 125 Worth Street, CN-34E, New York, NY 10013, USA; kito1@health.nyc.gov (K.I.); sjohnso5@health.nyc.gov (S.J.); eag2186@cumc.columbia.edu (E.A.G.); atang2@health.nyc.gov (A.T.); 2 Vital Strategies, 61 Broadway, Suite 2800, New York, NY 10006, USA; tmatte@vitalstrategies.org

**Keywords:** cold, hypothermia, cold-related illness, mortality, surveillance

## Abstract

Exposure to cold weather can cause cold-related illness and death, which are preventable. To understand the current burden, risk factors, and circumstances of exposure for illness and death directly attributed to cold, we examined hospital discharge, death certificate, and medical examiner data during the cold season from 2005 to 2014 in New York City (NYC), the largest city in the United States. On average each year, there were 180 treat-and-release emergency department visits (average annual rate of 21.6 per million) and 240 hospital admissions (29.6 per million) for cold-related illness, and 15 cold-related deaths (1.8 per million). Seventy-five percent of decedents were exposed outdoors. About half of those exposed outdoors were homeless or suspected to be homeless. Of the 25% of decedents exposed indoors, none had home heat and nearly all were living in single-family or row homes. The majority of deaths and illnesses occurred outside of periods of extreme cold. Unsheltered homeless individuals, people who use substances and become incapacitated outdoors, and older adults with medical and psychiatric conditions without home heat are most at risk. This information can inform public health prevention strategies and interventions.

## 1. Introduction

Environmental cold causes illness and death in many regions of the world [1,2,3,4,5]. Cold-related illness includes hypothermia, which occurs when the body’s temperature drops below 35 °C (95 °F), and less serious injuries, such as frostbite of the extremities [6]. Similar to heat exposure, cold temperatures can also result in illness and death by exacerbating chronic conditions, including respiratory and cardiovascular disease. 

Hypothermia deaths can occur at very low temperatures as well as temperatures as high as 22–24 °C (70–75 °F) among those who are older and medically compromised [6]. Similarly, some studies have found that mortality has a linear relationship with cold, with deaths occurring over a wide range of temperatures throughout the winter [1]. In addition, some studies have found that regions with moderate cold seasons have a higher burden of cold-attributable mortality [3]. Researchers have theorized that populations living in warmer, more variable climates may be less prepared for the cold with respect to indoor heating levels and personal precautions than those living in uniformly colder climates [2,5,7]. Other researchers, however, have found no difference in excess mortality burden by seasonal temperature range, theorizing that any differences detected are due to inadequate control for seasonal confounders [8].

Cold-related illness and deaths can be prevented with interventions at the individual, built environment, and community level [9]. For instance, individuals can wear protective clothing and stay in heated environments, social service agencies can conduct outreach to street homeless (i.e., unsheltered) individuals to encourage them to use shelters, and weatherization programs can help ensure that buildings retain more heat in the winter by assisting residents to better insulate their homes and increase energy efficiency. In the United States (US), the federal government provides grants to states, called the Low-Income Home Energy Assistance program, to help low-income residents with home heating costs if they meet certain criteria. In New York City (NYC), the US, building owners are legally required to provide heat to their tenants when the temperature drops to a pre-defined level. 

Despite warmer average global temperatures resulting from climate change [10], cold-related illness and death and winter excess mortality will continue to be health risks [11]. In NYC, climate change may also increase the frequency and severity of hurricanes, which in NYC can occur into the cold season, and increase the possibility of power outages due to high winds and storm surges, increasing the potential for exposure to cold conditions [12,13]. While individual events cannot be attributed to climate change, Hurricane Sandy provides an example of a storm that hit NYC at the beginning of the cold season, causing extensive flooding and utility outages that resulted in cold-related illness [14]. Few studies, however, have considered risk factors for cold-related illness and death to inform current and future response. 

The aim of this analysis was to understand the burden and risk factors for cold-related hospital admissions, emergency department (ED) visits and hypothermia deaths in NYC, which is home to 8.5 million people and is in the humid subtropical climate zone, though it borders the humid continental climate zone to the north of the city [15]. These deaths and illnesses represent a small fraction of the total burden of cold-related health impacts, which are largely comprised of exacerbation of chronic conditions and natural cause deaths, or “excess natural cause mortality,” which are not formally attributed to cold exposure. However, illness and deaths directly attributed to cold weather can be individually counted and investigated to describe risk factors for these health impacts. We examine cold-related illness and death, defined in this analysis as hypothermia and tissue damage due to environmental cold exposure, including a detailed investigation of medical examiner records for hypothermia deaths. This type of information can help elucidate risk factors not available in administrative data, such as whether decedents were exposed indoors or outdoors, had home heat, lived with others, and other circumstances of exposure. This information is crucial for designing and targeting public health interventions.

## 2. Materials and Methods

Treat-and-release ED visits (i.e., those that did not result in hospitalization) and hospital admissions for cold-related illness from 2005 to 2014 were examined in New York Statewide Planning and Research Cooperative System (SPARCS) hospital discharge data, a comprehensive dataset that includes patient-level data on all hospital visits in the state. Cases were defined as patients with any diagnosis code in the range of the International Classification of Diseases, Ninth Revision, Clinical Modification (ICD9-CM) 991 (“effects of reduced temperature”) or with an external cause of injury code E901.0, E901.8, E901.9, E988.3 (“excessive cold” or “extremes of cold” of unintentional or undetermined intent). Records with any diagnosis of E901.1 (“excessive cold of man-made origin”, for example exposure to dry ice) were excluded. NYC residents or potentially homeless individuals treated in NYC hospitals admitted during the cold season (October–April) were included. A residence indicator code noting whether a patient was homeless at the time of discharge was used to identify homeless individuals. 

Hypothermia deaths occurring in the cold season from 2005 to 2014 were examined using death certificate data provided by the NYC Department of Health and Mental Hygiene (DOHMH) Bureau of Vital Statistics. Cases were defined as deaths with an ICD-10 code of X31 (“exposure to excessive natural cold”) or T68 (“hypothermia”) as an underlying or contributing cause of death and excluded cases with any code of W93 (“exposure to excessive cold of man-made origin”) or codes indicating intentional deaths (X6–X9, Y0, Y35, Y36). Individuals who died in NYC and did not have an address were considered to be homeless. Because this dataset was not necessarily mutually exclusive with hospital visits, however, those who were admitted or died (*n* = 83) were excluded from ED visits. For hospital admissions, those who died were excluded (*n* = 353).

We stratified outcomes by age and gender because the existing literature on cold-related illness and death indicates that men are more at risk, as are the very young and old [6]. Demographic characteristics as well as place of injury and prevalence of other physical and mental health conditions were examined. Rate ratios were calculated using 2010 US Census data as denominators, and confidence intervals were calculated using a Poisson distribution. As a measure of deprivation, 2010–2014 American Community Survey data on the percentage of households by zip code living below the US federal poverty level were used to calculate rates by neighborhood poverty level. 

The burden of illness and death was estimated on extreme cold days, defined as days with minimum temperatures reaching −6 °C (20 °F) or below, approximately the 5th percentile for cold season minimum temperatures during the study period. We obtained weather data from the National Climatic Data Center for LaGuardia Airport, one of three NYC weather stations, selected because it had the most complete data. We estimated the burden occurring on extreme cold days at a 0–1 lag because previous analysis of hospital discharge data for cold-related illness in NYC indicated that the relationship with minimum temperature was largely limited to that period [16]. 

In NYC, the Office of the Chief Medical Examiner (OCME) investigates deaths suspected of being due to an external cause, including hypothermia deaths. For this analysis, OCME records were examined for a subset of deaths occurring from 2008 to 2013 to gather more information about circumstances of exposure and risk factors, including whether decedents were homeless or suspected to be homeless. Because this definition of cold-related death and homelessness is different from the NYC homeless surveillance system, death counts may differ from that surveillance system [17]. 

Body mass index (BMI) data calculated from the subset with OCME record review were compared with citywide BMI prevalence using self-reported data from the 2010 NYC Community Health Survey (CHS). Addresses of those exposed indoors or with unknown place of exposure were matched with city tax lot data to provide information about housing characteristics, and with 311 call data on complaints of lack of heat or hot water in the two weeks prior to the health event. Differences between BMI and housing characteristics were assessed with Chi-square and Fisher’s Exact tests.

The NYC DOHMH Institutional Review Board (IRB) determined that the OCME review was exempt research (#13068). A research agreement with the OCME allowed us to review records for hypothermia decedents identified in death certificate data. The NYC DOHMH IRB also reviewed and approved the study of hospital discharge and death certificate data (#14036).

## 3. Results

In NYC, on average during the ten-year study period, there were 180 treat-and-release emergency department visits (average annualized rate of 21.6 per million) and 240 hospital admissions (average annualized rate of 29.6 per million) for cold-related illness, and 15 cold-related deaths (average annualized rate of 1.8 per million) each cold season. There were 185 days (9% of all cold season days) when the current or previous day’s minimum temperature reached ≤−6 °C (20 °F). The mean daily minimum temperature was 4 °C (39 °F) and the mean daily mean temperature was 7 °C (45 °F). Thirty-two percent of deaths, 22% of admissions, and 30% of ED visits occurred on either the same day or the day following extreme cold (Table 1). 

### 3.1. Hospital Visits

The highest rates of ED visits occurred among men aged 35–64 years and those aged 85 and older (Table 1). About a third had one or more chronic conditions, most commonly substance use (15%). Among the 1101 (62%) with place of injury available, 36% were exposed on the street and 13% in a residence. 

There were more hospital admissions for cold-related illness than ED visits. Hospital admission rates increased with age, with the exception of children aged 0–4, who had higher rates than older children and young adults. Nearly all of those hospitalized (94%) had one or more chronic conditions, including cardiovascular disease (CVD) (61%), substance use (45%), mental illness (44%), respiratory conditions (31%), and diabetes (25%). Of those with place of injury information available (*n* = 1726, 71%), 35% were exposed on the street and 34% in a residence.

ED visits and hospital admission rates increased with neighborhood poverty (Table 1). A small number of both ED visits (*n* = 75, 4%) and hospitalizations (*n* = 94, 4%) had a complaint about no heat or hot water from the tax lot of residence in the two weeks prior to the visit. About a quarter of hospitalizations and 1% of ED visits were homeless people.

### 3.2. Deaths

In death certificate data, 72% had one or more co-morbidities, including CVD (38%), respiratory conditions (7%), and mental illness (5%, Table 1). Forty percent used substances. Decedents were disproportionately non-Hispanic black (*n* = 58, 39%, versus 23% from the 2010 Census). More deaths occurred in residents of medium-, high- and very-high-poverty neighborhoods compared to low-poverty neighborhoods (Table 1). The average mean temperature on the day of death was 1 °C (range: −13, 19 °C) [34 °F, range: 9, 66 °F], compared with 7 °C (range: −13, 26 °C) [45 °F, range: 9, 79 °F] for the cold season generally. 

Among the subset reviewed in medical examiner records (*n* = 76, Table 2), 96% had one or more comorbidities, including CVD (74%), evidence of alcohol use at time of death (34%), and cognitive or mental health conditions (29%). Decedents were significantly more likely to be normal or underweight than the general NYC population (58% versus 43%, *p* ≤ 0.001). More than one third of decedents were homeless or suspected to be homeless (*n* = 30, 39%). Compared to the total sample, more homeless decedents were men (87% versus 70%), and the percentage of decedents with normal or underweight BMI was similar (56% among homeless versus 58% among the total sample).

Most decedents (*n* = 57, 75%) were exposed outside (Table 2). All homeless individuals were exposed outside, representing about half of outdoor exposures. Most of the remaining outdoor exposures involved alcohol or substance use. The most common co-morbidities among people exposed outside were CVD (67%), chronic or acute substance use (61%), and cognitive or mental health conditions (16%). 

Of those exposed indoors (*n* = 19, 25%), none had home heat (Table 3). All were aged 60 or older. More than half had a mental health or cognitive condition (*n* = 13, 68%). Records noted evidence of hoarding for more than a third (*n* = 7, 37%). Detached single-family homes were the most common housing type. The prevalence of this type of housing among decedents was significantly greater than the prevalence of single-family homes in NYC generally (67% versus 16%, *p* ≤ 0.001). Of those with information on other residents living in the home (*n* = 16), nearly half (*n* = 7, 44%) lived alone. Of the 9 individuals living with others, two pairs of people died together. One complaint about no heat came from a tax lot of a decedent within two weeks prior to the death.

## 4. Discussion

This study combined hospital discharge and vital statistics data with the collection of data from medical examiner records to characterize the burden of, and risk factors for, cold-related illness and death in the largest city in the United States. Rates of illness and death were higher among older adults, men, non-Hispanic black individuals and those living in higher-poverty neighborhoods, consistent with US national studies of hypothermia deaths [18]. Most decedents (75%) were exposed outdoors and, of those exposed outdoors, about half were homeless. Of decedents exposed indoors (25%), none had home heat, nearly all were living in single-family or row homes, and 68% had a mental health or cognitive condition. 

Similar to those at risk for serious heat-related illness or death in NYC, people who died or were hospitalized for cold-related illness were older and had multiple chronic conditions when compared to patients treated and released from emergency departments. The most common co-morbidities among decedents and those hospitalized included CVD, substance use, and mental illness. Also similar to heat-related deaths in NYC, non-Hispanic black individuals were at increased risk, likely attributable to the effects of structural racism on health and income inequities [19]. 

There were also differences when compared to risk factors for heat-related illness. There were fewer cold-related ED visits on average each year than for heat-related illness [20] but more hospitalizations and deaths, consistent with US national studies [21]. The higher admission rate may be in part because homeless adults are disproportionally hospitalized in NYC, potentially because of a lack of discharge options [22]. It is also possible that heat ED visits include more exertional illness (for instance, related to sports or work) than cold, which may be easier to treat without needing to admit the patient. In addition, compared with heat-related hospital visits in NYC [20], the age distribution of patients treated with cold exposure is generally older, with the male aged 35–64 group contributing the largest proportion (approximately 40%) for both ED visits and hospitalizations. Younger people tend to have fewer co-morbid conditions, and may be more likely to recover from exertional heat stress with hydration and be less likely to need admission. For both heat and cold, however, ED visits coded as work-related are relatively small (7% for heat and 3% for cold, data not shown). 

Furthermore, unlike hyperthermia decedents in NYC, the majority of whom are exposed indoors, most who died of hypothermia were exposed outdoors—about half of those exposed outdoors (30 decedents out of 57 exposed outdoors) were homeless. In addition, decedents were more likely to be normal or underweight, whereas hyperthermia decedents were more likely to be overweight or obese than NYC adults generally [20]. Finally, most hyperthermia deaths in NYC are associated with extreme heat events [20], while most cold-related deaths occurred on days that were colder than average for the season but not extremely cold. This underscores the importance of preventing exposure even when temperatures are not extreme. Additional research could further assess the relationship between cold-related illness and death and weather conditions, as well as the relationship between cold-related illness and death and excess natural cause mortality. More research should also assess the relationship between temperature and cold-related illness and death, including an assessment of the potential impact of future climate change on these outcomes.

Ensuring home heat among vulnerable people also remains an important strategy to prevent serious illness and death. Of decedents exposed indoors, none had home heat. Nearly all lived in single-family homes and may have been unable to pay heating costs. A previous study of indoor temperatures in NYC found that single-family detached homes were the building type that would most rapidly lose heat in cold weather without heating, also indicating that this is a particularly risky form of housing in which to lack heat [23]. A small percentage of patients treated in the ED or hospitalized had made a telephone call to the city’s toll-free service for urgent complaints (locally known as “311”) from their residential tax lot, suggesting that lack of home heat can be a problem for those with non-fatal cold-related illness as well.

While mental illness was only listed in 5% of hypothermia death certificate records, it was noted among 29% of decedents in medical examiner records, highlighting an advantage of utilizing these more detailed records. Mental health conditions were much more common among those exposed indoors (68%), including many with a history of hoarding (37%). One Australian study of deaths among hoarders found evidence of hypothermia in 15% of decedents, noting that they may have lacked heat, potentially impeded by extensive clutter [24]. The study also found that hoarding decedents overall were socially isolated, in poor health, and often used alcohol. People with schizophrenia and other mental health conditions may be at increased risk of cold-related illness and death for a number of reasons, including taking psychiatric medications that can impair thermoregulation [6], cognitive impairments that can affect the ability to take protective actions, social isolation, and a higher prevalence of health co-morbidities.

Among decedents for whom medical examiner records were reviewed, there was a higher prevalence of substance use (chronic or acute) for those exposed outdoors (61%), and only 16% had a history of cognitive or mental health conditions. Given that half of decedents exposed outdoors were homeless, however, the lower prevalence of mental health conditions among decedents exposed outdoors may be a result of a lack of available medical and psychiatric history, rather than an absence of conditions. Research has shown that serious mental health conditions are more common among homeless individuals than the general population [25]. A study of homeless health from 2001 to 2003 also found that nearly 40% of cold exposure decedents in NYC were homeless, with alcohol and substance use and CVD as important contributing factors [22]. Findings that homeless individuals were all exposed outdoors is also consistent with previous research on mortality among the homeless in NYC, which found that hypothermia decedents were all street (i.e., unsheltered) homeless individuals [17]. Another study of large urban areas of Poland also found that homeless individuals died of hypothermia in both extreme and moderate cold periods, highlighting the need for further research on potential temperature thresholds for cold-related health impacts in this population [26].

There are several limitations to this analysis. Deaths and hospital visits from chronic conditions, such as CVD, exacerbated by cold are not included, so this is not a complete assessment of the health impacts of cold weather. Similar to hyperthermia deaths, which are much fewer in number than heat-related natural-cause mortality [27], hypothermia deaths alone are an underestimate of the true burden of cold mortality. Examining illness and death directly attributed to cold, however, allows more detailed examination of risk factors. Another important limitation is that, because notes in medical examiner records and lack of address on death certificates were used to characterize homelessness among decedents, there may be misclassification and we were likely unable to fully capture homeless status [28]. Administrative data sources such as hospital discharge and vital statistics data likely underestimate the number of homeless individuals. In addition, removing patients who died from hospital discharge counts may help to avoid potential double-counting with death records, but may also result in an underestimate of cases. The hospital discharge and death certificate datasets were not linked, so patients with a cold-related illness code who were noted to have died in hospital data may be different from hypothermia decedents in death certificate data. Place of injury in hospital discharge data was also frequently missing. Finally, the case definition included 1 death, 8 ED visits, and 10 admissions that had solely a code for cold exposure of undetermined intent. Despite these limitations, we have been able to use relatively complete data, including detailed case investigations, to characterize the burden of serious illness and death directly attributable to cold in NYC.

## 5. Conclusions

In NYC, unsheltered homeless individuals, adults using substances who become incapacitated outdoors, and older adults with mental health conditions living in detached single-family homes without heat are at risk of cold-related death and illness. Outreach to street homeless individuals can help prevent illness and death. Public health messaging should target those most at risk and should include information about financial resources for home heating, such as the Low-Income Home Energy Assistance Program, particularly for socially isolated older adults living in private homes who may be responsible for heating costs. Although the climate is warming, cold exposure is an ongoing concern during the winter. Further research should assess the relationship between weather conditions and cold-related illness and deaths, including whether that relationship differs by homeless status. More research is also needed on cold weather and natural cause mortality under current and future climate conditions.

## Figures and Tables

**Table 1:** Characteristics of New York City (NYC) residents treated for cold-related illness or who died as a result of hypothermia, 2005–2014.

Patient Characteristic	Emergency Department Visits, Excluding Admissions and Deaths				Hospital Admissions, Excluding Deaths				Deaths			
	*n*	%	Rate Ratio	95% CI	*n*	%	Rate Ratio	95% CI	*n*	%	Rate Ratio	95% CI
Total ^1^	1768	100%	-	-	2419	100%	-	-	148	100%	-	-
Gender												
Female	504	29%	ref	-	770	32%	ref	-	44	30%	ref	-
Male	1263	71%	2.8	2.5, 3.1	1649	68%	2.4	2.2, 2.6	104	70%	2.6	1.8, 3.7
Unknown ^2^	1											
Female age group (years)												
0–4	19	1%	ref	-	22	1%	ref	-	-	-	-	-
5–17	70	4%	1.5	0.9, 2.5	16	0%	0.3	0.2, 0.6	1	1%	-	-
18–34	117	7%	1.3	0.8, 2.2	44	2%	0.4	0.3, 0.7	1	1%	-	-
35–64	219	12%	1.8	1.1, 2.8	259	11%	1.8	1.2, 2.8	17	11%	ref	-
65–84	59	3%	1.6	0.9, 2.6	268	11%	6.2	4.0, 9.5	16	11%	3.1	1.6, 6.2
85+	20	1%	2.7	1.4, 5.1	161	7%	18.9	12.1, 29.5	9	6%	9.0	4.0, 20.1
Male age group (years)												
0–4	23	1%	ref	-	36	1%	ref	-	-	-	-	-
5–17	87	5%	1.6	1.0, 2.5	16	0%	0.2	0.1, 0.3	-	-	-	-
18–34	332	19%	3.5	2.3, 5.3	151	7%	1.0	0.7, 1.5	6	4%	ref	-
35–64	704	40%	5.4	3.6, 8.2	1028	42%	5.1	3.6, 7.1	67	45%	8.2	3.6, 18.9
65–84	102	6%	3.3	2.1, 5.2	345	14%	7.2	5.1, 10.2	26	18%	13.5	5.6, 32.8
85+	15	1%	4.0	2.1, 7.6	73	3%	12.4	8.3, 18.5	5	3%	21.1	6.5, 69.3
Neighborhood Poverty ^3^												
Low (<10% in poverty)	198	11%	ref	-	272	11%	ref	-	11	9%	ref	-
Medium (10 to <20%)	573	33%	1.4	1.2, 1.6	873	37%	1.5	1.3, 1.7	50	42%	2.1	1.1, 4.1
High (20 to <30%)	470	27%	1.7	1.4, 2.0	592	25%	1.5	1.3, 1.8	28	23%	1.8	0.9, 3.6
Very High (30%+)	502	29%	2.0	1.7, 2.3	647	27%	1.9	1.6, 2.1	31	26%	2.2	1.1, 4.4
Other/Unknown ^2^	25				35				28			
Homeless ^4^	15	1%			586	24%			26	18%		
Other Health Conditions ^5^												
Cardiovascular	173	10%			1478	61%			56	38%		
Substance Use/Dependency	272	15%			1099	45%			59	40%		
Mental Illness	86	5%			1075	44%			8	5%		
Respiratory	66	4%			752	31%			10	7%		
Diabetes	86	5%			611	25%			6	4%		
Any condition	536	30%			2269	94%			107	72%		
Place of Injury												
Residence	142	13%			580	34%						
Street	397	36%			611	35%						
Recreation	31	3%			36	2%						
Building	28	3%			36	2%						
Other	503	46%			463	27%						
Unknown ^3^	667				693							
Current or previous day min temp ≤ −6 °C (20 °F)	534	30%			533	22%			47	32%		

^1^ Data restricted to events in months of October–April. Rate ratios calculated using 2010 US Census data for denominators; ^2^ Excluded from denominator for percentages; ^3^ Neighborhood poverty based on zip code defined as percent of residents with incomes below 100% of the Federal Poverty Level per the US American Community Survey 2010–2014; ^4^ Based on homeless indicator in hospital data and residence unknown in death certificates; ^5^ Not mutually exclusive. Any chronic condition defined as having one or more of the following conditions: cardiovascular disease, substance use, mental illness, respiratory conditions, or diabetes.

**Table 2:** Selected medical and social characteristics of decedents based on a review of NYC hypothermia fatalities by place of onset, 2008–2013.

Patient Characteristic	Total		Onset Indoors		Onset Outdoors ^1^	
	*n*	%	*n*	%	*n*	%
Total	76	100%	19	100%	57	100%
One or more chronic condition(s) ^2^	73	96%	19	100%	54	95%
Evidence of cardiovascular disease	56	74%	18	95%	38	67%
History of diabetes	9	12%	3	16%	6	11%
Evidence of a respiratory condition	6	8%	1	5%	5	9%
Substance Use						
Evidence of alcohol (chronic or acute) or other substance use	40	53%	5	26%	35	61%
Evidence of alcohol use at time of death	26	34%	1	5%	25	44%
Cognitive or mental health conditions						
Any cognitive or mental health condition ^3^	22	29%	13	68%	9	16%
History of schizophrenia/schizo-affective disorder	8	11%	2	11%	6	11%
History of hoarding	7	9%	7	37%	0	0%
Body Mass Index category (18 years of age and older, *n* = 75)						
Normal or Underweight	43	58%	10	53%	33	60%
Overweight	20	27%	5	26%	15	27%
Obese	11	15%	4	21%	7	13%
Unknown ^4^	1				1	
Homeless ^5^	30	39%	0	0%	30	53%

^1^ Includes those with onset at subway stations or abandoned buildings; ^2^ Records with any evidence of cardiovascular disease, substance use, diabetes, cognitive or mental health condition, or respiratory condition. Does not include body mass index; ^3^ Including schizophrenia and hoarding; ^4^ Excluded from denominator for percentages; ^5^ Homeless status determined by mention of confirmed or suspected homelessness in NYC Office of Chief Medical Examiner records, including death certificates, case worksheets, and investigation reports.

**Table 3:** Selected housing characteristics of decedents based on a review of NYC fatalities with residential onset, 2008–2013 (*n* = 19).

Housing Characteristics	Decedents		NYC Housing	
	*n*	%	*n*	%
Home heat				
No home heat	19	100%	n/a	
Residence type ^1^				
Single-Family	10	67%	539,540	16%
Row House	3	20%	520,031	15%
Brick Lowrise	1	7%	910,452	27%
Highrise	1	7%	1,435,246	42%
Other/Unknown ^2^	2		807	

^1^ Includes prevalence of housing type among decedents and among the general NYC population, rather than the percentage of people living in each type of housing. The data source for overall NYC housing is the NYC Property Land Use Tax lot Output (PLUTO); ^2^ Excluded from denominator for percentages.
